# Distinguishing synaptic vesicle precursor navigation of microtubule ends with a single rate constant model

**DOI:** 10.1038/s41598-021-82836-7

**Published:** 2021-02-09

**Authors:** M. W. Gramlich, S. Balseiro-Gómez, S. M. Ali Tabei, M. Parkes, S. Yogev

**Affiliations:** 1 Department of Physics, Auburn University, Auburn, AL USA; 2 Departments of Neuroscience and Cell Biology, Yale School of Medicine, New Haven, CT USA; 3 Department of Physics, University of Northern Iowa, Cedar Falls, IA USA

**Keywords:** Biophysics, Cytoskeleton, Endosomes, Biological physics

## Abstract

Axonal motor driven cargo utilizes the microtubule cytoskeleton in order to direct cargo, such as synaptic vesicle precursors (SVP), to where they are needed. This transport requires vesicles to travel up to microns in distance. It has recently been observed that finite microtubule lengths can act as roadblocks inhibiting SVP and increasing the time required for transport. SVPs reach the end of a microtubule and pause until they can navigate to a neighboring microtubule in order to continue transport. The mechanism(s) by which axonal SVPs navigate the end of a microtubule in order to continue mobility is unknown. In this manuscript we model experimentally observed vesicle pausing at microtubule ends in *C. elegans*. We show that a single rate-constant model reproduces the time SVPs pause at MT-ends. This model is based on the time an SVP must detach from its current microtubule and re-attach to a neighboring microtubule. We show that vesicle pause times are different for anterograde and retrograde motion, suggesting that vesicles utilize different proteins at plus and minus end sites. Last, we show that vesicles do not likely utilize a tug-of-war like mechanism and reverse direction in order to navigate microtubule ends.

## Introduction

The length of neuronal axons necessitates a dedicated transport system to deliver cargo from the cell body over large distances. Molecular motors dynein^1^, and kinesin superfamily members^2^, use the uniform plus-end-out microtubule (MT) array of axons as a substrate on which to transport axonal cargo retrogradely or anterogradely, respectively. Axonal transport is crucial for neuronal viability, and its dysfunction is a hallmark of neurodegenerative diseases^3^.

Paradoxically, despite the need to cover long-distances, cargo motility in axons is interspersed with frequent pauses^4,5^. The fraction of time cargo spends being immotile varies considerably among cargo types and experimental systems^6,7^. Explanations for these stalls include a tug-of-war between dynein and kinesin motors^8,9^, interactions with physical barriers (such as other organelles, MAPs or actin)^5,10^, and pauses at MT ends.

Tug-of-war mechanisms have been extensively studied in vitro^9^, and in vivo^8^. Protein barriers have been likewise studied in vitro^11^, and in vivo^12^. *However*, cargo pauses at MT-ends along the axon have been less well studied with in vitro studies of structural MT-defects have focused exclusively on single MTs^13^. Our recent in vivo experiments of synaptic vesicle precursors (SVPs) have shown a significant inhibitory effect at MT-ends^7^. We focus here on understanding the mechnism by which recently observed in vivo SVPs navigate MT-ends^7,14^.

All axons harbor parallel tracks of individual MT polymers, which are much shorter than the axon itself^7,15–19^. When a cargo reaches a MT-end it must somehow detach and re-attach somewhere else in order to navigate the MT-end. This raises the question of how the cargo negotiate the transfer from one polymer to the next at MT ends. Our previous work revealed that in *C.elegans* motor neurons, most pauses during the transport of SVPs occur at MT tips, indicating that negotiating the transfer from one polymer to the next is rate-limiting for efficient transport in this system^7^. This conclusion is consistent with cell-culture and in vitro studies in which MT ends inhibit motility of kinesin-3 and dynein motors, which drive anterograde and retrograde SVPs transport in neurons^14,20–22^.

Several possible scenarios may occur for SVPs at microtubule ends: (i) vesicle complexes may fall off at the MT-end, then the vesicle diffuses until it binds a new motor somewhere else along the axon and become motile again; (ii) multiple motors bound to the vesicle complexes may compete at a MT end, and the resumption of vesicle motility would depend on the paused motor dissociation from the MT-end. This scenario is consistent with the notion that SVPs are likely transported by several motors simultaneously^23^; (iii) the vesicle complex detaches from the MT at the end and the same motor-vesicle reattaches to another MT along the bundle.

Studies in vitro and in non-neuronal cultures have partially addressed these possibilities. For example, dynein motors show tenacious binding of MT-minus-end at the end of their runs in vitro^20,21^. Conversely, kinesins display a more variable behavior at the plus-end, ranging from tight binding to falling off^14,22,24^, which depends on the specific type of kinesin examined, the presence of co-factors, the GTP-state of the MT and the experimental system. However, analyzing similar events in neurons faces two significant technical obstacles: (1) The difficulty of visualizing individual motors limits the analysis to the motility of cargo and (2) The high density of MTs in axons limits any ability to resolve which polymer a given SVP is associated with at the light-microscopy level. Hence, it is presently unknown how SVP negotiate microtubule ends to resume motility in neurons. One approach that could bridge the gap between in vitro observations and studies in living neurons is modeling.

Here we develop a simple probabilistic model for the behavior of motor-SVP complexes at MT ends during axonal transport. Importantly, the model enables us to quantitatively describe the observed pausing behavior using a single parameter. We then test the model against in vivo live imaging of SVP transport in *C.elegans* motor neurons, where we have shown that pauses during transport occur at MT ends. We find that the model reliably explains the behavior of vesicle at MT ends. These studies provide insight into long-range transport of synaptic cargo in axons.

## Results

### Quantifying pause times at microtubule ends

**Figure 1:**
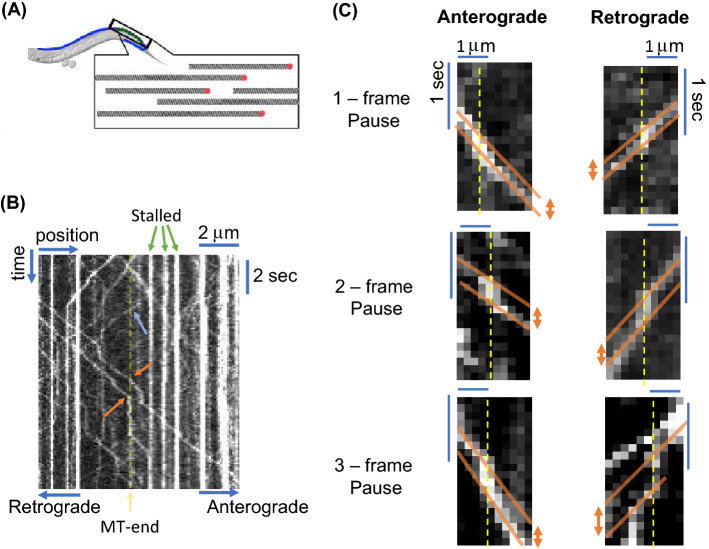
MT-end kymograph analysis method. (**A**) Cartoon geometry of *C. elegans* axon and microtubule network. The axon has multiple tracks of microtubules aligned parallel to each other with breaks between individual microtubules. Figure reproduced with permission from Yogev et al.^7^. (**B**) Kymograph (time/space) representation of SVPs navigating an axon. A microtubule end is designated as dashed-yellow line (determined by fraction of traverses as described in 2.2). Vesicles are marked as traversing (blue arrow) or pausing (orange arrow) at the end. Vesicles engage in both anterograde and retrograde motion. (**C**) Examples of quantification of short-time vesicle pausing at microtubule-ends (dashed yellow lines determined by fraction of traverses as described in 2.2). The pause-time at a MT-end is determined as the time difference between velocity traces (orange solid lines). Both anterograde and retrograde motion are quantified the same and exhibit the same pausing behavior.

We first characterized MT-end locations along an axon in order to determine pause-times (Fig. 1, Supplementary Movie 1). SVPs were labelled by the canonical marker GFP::RAB-3 and displayed the predicted pattern of presynaptic varicosities as previously described^25,26^. SVPs exhibited the ability to both traverse (blue arrow in Fig. 1B) and pause (orange arrow in Fig. 1B) at the same x-axis location along a kymograph. In order to determine locations of MT-ends at x-positions, we identified positions where SVP transport frequently paused, as our previous work identified these locations as microtubule ends^7^. We required *at least* 10 vesicles observed to traverse and *at least* 5 vesicles were observed to pause. The combined number of traverse and pause SVPs would give an uncertainty in measuring any given pause-time of < 10% for each MT-end measured (See Appendix A1 for methods).

We noted that some locations also had SVPs stalled before imaging began and remained at the same location for the entire experiment (Green arrows Fig. 1B). Although such locations likely correspond to MT ends^7^, we sought to eliminate any possible effect of vesicle aggregation^27^ or other regulatory mechanisms that may retain cargo in these locations. Our focus in this work was on the role of MT-ends and not stalled vesicles. Any motile vesicle, which paused at the same location as a stalled vesicle, may have paused because of the stalled vesicle and not the MT-end. The imaging resolution could not distinguish between a pause caused by a MT-end and a stalled vesicle. Thus, we did not include locations that had vesicles paused before imaging began.

We quantified SVP pausing at MT-ends based on velocity and time-shifts in kymograph data (Fig. 1C). We particularly focused on short-time pauses because they represented the majority fraction of all vesicle pausing, especially at sites that did not contain stalled SVPs before imaging began. We distinguished between pause times by following protocol: (i) we calculated the slope of vesicle trajectory before (solid orange line in all figures) reaching a known MT-end location (dashed yellow line in all figures); (ii) we then calculated the slope of vesicle trajectory after the known MT-end location (solid orange line in all figures); (iii) we then determined the intercept for each trajectory either before (for anterograde motion) or after (for retrograde motion) at a fixed position (slopes are drawn to the edge of the kymograph where they are compared in Fig. 1C); (iv) finally, the difference between intercepts is then the pause time for the vesicle. Using this method we can easily distinguish between short-time (1–3 frames) pauses as shown by the examples in Fig. 1C.

We note that as controls for MT-end locations, we also chose random locations along the x-axis where at least 20 SVPs observed to traverse but no requirement was set for the number of vesicle pauses at that location. We used the same quantification method to determine short-time pauses at these random locations. We chose the same number of locations as MT-end locations. Last, we followed the same data aggregation method for these random locations in order to determine pause-time distribution (See Appendix A1).

### Probability of traversing a MT-end

We first quantified the fraction of attempts that motor driven SVPs walk past a MT-end. If a vesicle is on a MT that ends, then it must detach and re-attach to another MT along the axon. However, in vivo fluorescence microscopy experiments cannot distinguish which MT a vesicle is on. Thus, if a vesicle is travelling along the axon, fluorescence experiments measure the probability that it is on the MT that ends. This probability is simply the inverse of the number of laterally aligned MTs. This probability is true regardless of the motor or direction of travel. Thus, we quantified the probability that vesicles *walk past* a MT-end in order to determine if simple probabilities dictate motor pausing. It is important to note that all MTs along an axon have their polarities aligned with plus-end distal and minus-end proximal oriented^17,28,29^.

Experimentally we observed that both retrograde (Dynein) and anterograde (kinesin-3/UNC-104) driven motion exhibit similar probability of traversing MT-ends. Retrograde driven vesicles traversed MT-end locations the majority of times observed (0.7345 ± 0.02). Anterograde driven motion also traversed the same MT-end locations the majority of times observed (0.7299 ± 0.02). Both anterograde and retrograde exhibited the same traversal fractions and were not statistically significantly different (P = 0.9112, two-tailed Students t-test). The overall fraction of vesicle traversals suggest that vesicles have approximately 1 in 4 probability of being on a MT with a MT-end, which is consistent with the range of MT tracks in the DA9 axon, as previously determined by EM reconstructions^7^.

**Figure 2:**
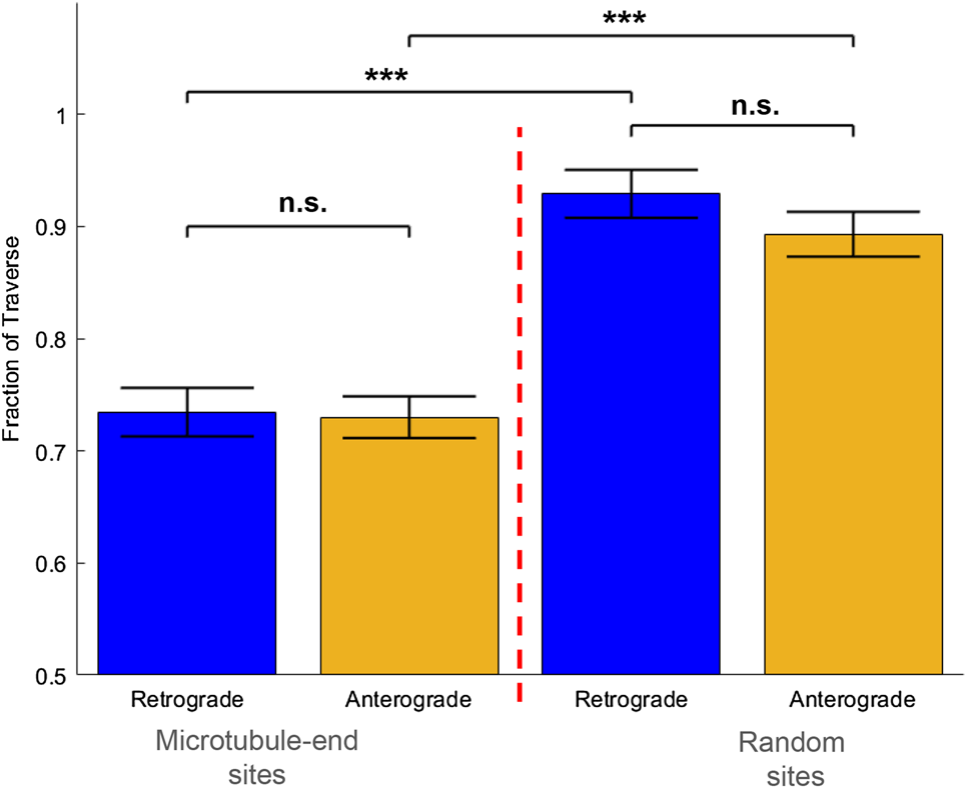
Probability Vesicles Traverse A MT-end. Both retrograde (Blue Bars) and anterograde (Yellow Bars) driven vesicles traverse MT-ends for the majority fraction of attempts (> 0.7 for both). Retrograde and anterograde motion traverse a greater amount at randomly chosen axon locations, as compared to MT-end locations. *** = p < 0.01; Error-bars are determined by standard-deviations in fraction of observed traversals per location.

We controlled for random pausing effects by comparing traverse fractions at MT-ends to traverse fractions at randomly chosen locations along the axon (Fig. 2). If vesicle traversing at MT-ends is unrelated to MT-end locations, then vesicles would exhibit the same fraction of traversals at randomly chosen locations. However, there is a statistically significantly higher rate of vesicle traversals at random locations. Both retrograde driven motion (MT-end to random location; P = 5.06E−5; two-tailed t-test) and anterograde driven motion (MT-end to random location; P = 9.24E−6; two-tailed t-test) exhibit the same higher traversals (Retrograde: 0.92 ± 0.02; Anterograde: 0.89 ± 0.02; p = 0.1146; two-tailed t-Test).

### Vesicle reversals suggest minimal tug-of-war motion

We next turned to distinguishing the dominant mechanism by which SVPs navigate MT-ends. There have been multiple proposed models axonal cargo *in general* use to navigate the cytoskeleton such as: (i) the “bucket-brigade” model^30^, which suggests motors are constantly binding and detaching from cargo but directed motion continues as if a single motor drives mobility; (ii) multiple bound motors per cargo coordinate motion^31,32^; (iii) a “tug-of-war” model between anterograde/retrograde motors where cargo will occasionally engage both motors simultaneously to navigate obstructions^4,9,33^. Distinguishing between different models is essential to understand the specific mechanisms by which SVPs use to navigate MT-ends.

**Figure 3:**
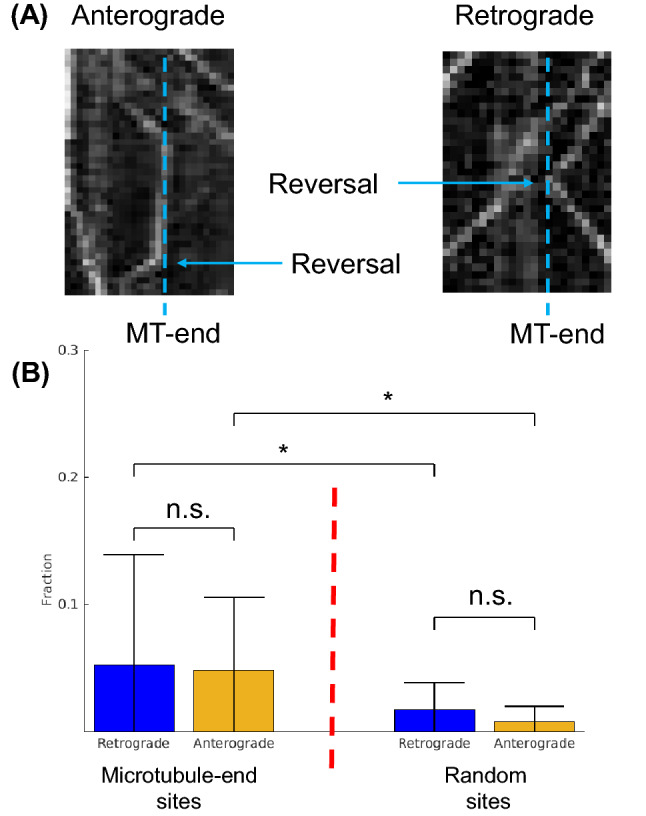
Vesicle reversal fraction *after* pausing at a microtubule-end. (**A**) Both anterograde and retrograde motion exhibit reversals at a microtubule-end location. Reversals are not observed to correlate with pause time. (**B**) The overall fraction of reversals at microtubule-ends is < 0.05 for both retrograde and anterograde motion. There is no statistical difference for direction of motion at MT-end. The overall fraction of reversals at random locations along the axon is < 0.02 for both retrograde and anterograde motion. There is no statistical difference for direction of motion at random locations. Error-bars are determined by standard-deviations in fraction of observed traversals per location. * = p < 0.05, two-tailed student t-Test.

We first focused on whether the “tug-of-war” mechanism was involved in SVP motion by quantifying vesicle reversals. Tug-of-war was first observed in vesicles that reverse direction at random locations along the axon. This observed tug-of-war behavior was distinguished from the same motor reversing direction due to oppositely polarized MTs because axons have all MTs oriented with the same polarity^17,28,29^. We observe that vesicles engage in reversals at MT-end locations (Fig. 3A). Indeed, both anterograde and retrograde motion exhibit reversals at MT-ends. *If SVPs utilize the “tug-of-war” mechanism then vesicles reversals at MT-ends would be a significant fraction of overall observed motion. Alternatively, if “tug-of-war” does not significantly contribute to how SVPs navigate MT-ends then reversals would be a small fraction of vesicle motion.*

We observed that vesicle reversal is a small portion of overall vesicle mobility at MT-ends (Fig. 3B). Importantly, reversals are not observed to correlate with pause time (Data not shown). We determined aggregate vesicle reversals by counting reversals across 34 MT-ends in 8 different experiments and divided by the total number of tracks (estimated from the traverse frequency) for the same MT-end. Both Retrograde and anterograde motion exhibited the same overall low reversal fraction with no statistically significant difference (Retrograde = 0.05 ± 0.086, Anterograde = 0.048 ± 0.057; P = 0.6255 from 2-tailed t-test). Thus, reversal and any potential motor transition at MT-ends is a low fraction of overall SVP mobility at MT-ends.

We compared the fraction of observed vesicle reversals at MT-ends to observed vesicle reversals at random locations along the same axons. We determined aggregate vesicle reversals by counting reversals across 34 locations in 8 different experiments and divided by the total estimated number of tracks for the same locations. Reversals at random positions along the axon occurred in less than 2% of all observed pauses for both retrograde and anterograde motion (Retrograde = 0.017 ± 0.02, N = 599; Anterograde = 0.008 ± 0.01, N = 737; P = 0.182 from 2-tailed t-test). There was a small but significant increase in reversals at MT-ends compared to random locations for both retrograde (P = 0.0463 from 2-tailed t-test) and anterograde (P = 0.0223 from 2-tailed t-test) motion, consistent with the notion the MT-ends impinge on vesicle transport. However, the overall scale of reversals regardless of location is small compared to the total number of observed vesicles.

The small fraction of reversals suggests that *the “tug-of-war” mechanism is minimally utilized by vesicles to navigate MT-ends*.

### Single rate-constant model of SVP navigation of MT-ends

The low-reversal results lead to the following question: *what determines how long a SVP will pause at a MT-end before resuming mobility?* If vesicles do not reverse, then what ever proteins driving the vesicle before the MT-end may likely dominate the time required for a vesicle to navigate the MT-end.

We propose the following hypothesized model: SVP navigation of MT-ends depends on the direction currently used by the vesicle when it encounters a MT-end. This hypothesis suggests that the time paused at MT-ends depends on the way in which the specific motors driving SVP motility prior to arrival at the tip interact with the MT-end structure or MT-end resident proteins. Note that given the unique structure of MT-ends, attachment/detachment rate constants at these locales would differ from the rate constants measured along the microtubule lattice in vitro (See discussion section).Further, this hypothesis suggests that anterograde and retrograde motion should exhibit different distributions of pause-times at MT-ends.

We now propose a model and method to distinguish our hypotheses by which vesicles navigate MT-ends. This model is based on two observations/assumptions: (i) The probability that a SVP will pause is only dependent on the microtubule track it is currently attached (based on the traverse fraction in Fig. 2); (ii) The time an SVP pauses involves any multi-protein-MT-end detachment/attachment rate-constants of the vesicle is utilizing when it reaches a MT-end. With these two observation-based assumptions we created a probabilistic model and computational simulation similar to observed vesicle transport.

**Figure 4:**
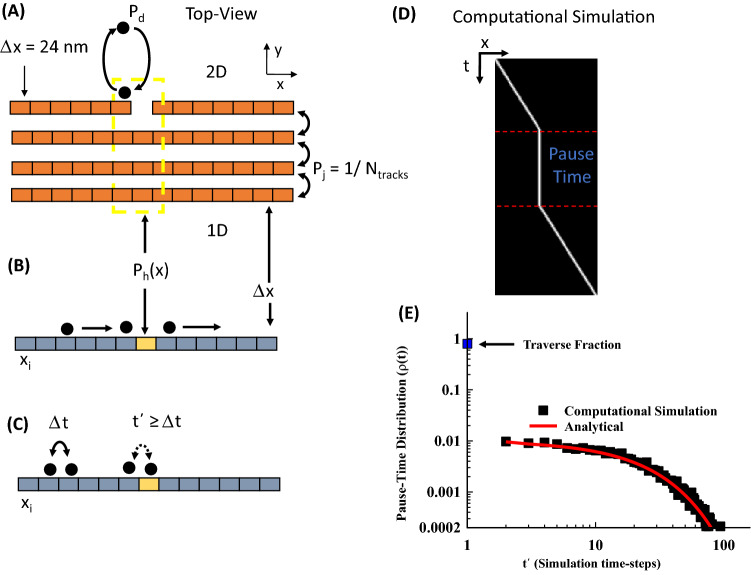
Dominant Motor-driven model. (**A**) We model an axonal bundle as lattice sites along a series of parallel tracks. Vesicles are modeled with a single detachment/attachment parameter (Pd). The probability a motor will jump between tracks is modeled as a uniform probability (Pj). MT-ends are modeled as missing lattice sites. (**B**) We reproduce kymograph results by making a one-dimensional simplification of our two-dimensional model. Each one-dimensional lattice site has the same spatial resolution as the two-dimensional lattice site (Δx). Vesicle inhibition by a MT-end site is now modeled as a probability (Ph), due to the loss of information about which track the vesicle is on. (**C**) The time a simulated SVP pauses at a location with a MT-end (t’) is greater than the time required to transition to the next lattice site (Δt). (**D**) A kymograph representation of a simulated vesicle pausing at the an x-lattice site, but with the exact track location is lost. (**E**) We introduce the Pause-time parameter to quantify and measure the probability a vesicle will pause at a MT-end. We show that our detachment parameter hypothesis (Red-line, Eq. 1) will show a uniform decrease in pause-time with increasing time. We compare our analytical expression with a computational simulation (Black-squares, 1000 simulations).

It is important to note that we only compare the times our simulated cargo spend at MT-ends to observed experimental times, however, we model transport along the entire axon to ensure that our model is also consistent with observed experimental kymograph results (Kymograph Fig. 4D).

#### Axonal bundle modelling

We modeled the axon as a bundle of aligned microtubules tracks^17^, as observed experimentally (Fig. 1A). The tracks are a two-dimensional arrays with rows representing tracks of individual microtubules and columns representing a coarse-grained lattice site (Fig. 4A). Microtubules are aligned along tracks equidistant from each other in a radial fashion similar to a neuronal axon or dendrite^34^. We set the minimum spatial resolution limit of our model to the size of a microtubule end, equal to the diameter of a single microtubule ~ 24 nm (represented by boxes in Fig. 4A)^35,36^. Single microtubules of finite-length populate these tracks with MT-ends represented by a single missing lattice site (Fig. 4A).

#### Vesicle transport modelling

We model vesicle transport as a discrete process, similar to a method previously used for single motor motility^11^, with a *single rate* process modeling overall SVP mobility. In this model vesicle transport mechanics are coarse grained to a single rate-constant parameter, we call *pause-duration* (P_d_, Fig. 4A). This parameter represents vesicle pausing as a discrete finite probability threshold that includes: (i) vesicle detachment time from the microtubule; (ii) vesicle diffusion to the same or another MT; (iii) vesicle re-attachment time to a MT. All three processes occur on time-scales less than experimentally observable, including vesicle diffusion between tracks^37^, and thus can be coarse-grained into a single parameter without losing accurate representation of experimentally observed results, which we show below.

Vesicles that detach at a MT end can hop between microtubule tracks in the bundle and reattach on any microtubule within the bundle (Fig. 4A). The probability of hopping on any microtubule track is equal for all tracks in the bundle (Pj = 1/Ntracks, where Ntracks is the number of tracks in a bundle). This equal weighting is assumed for simplicity in our model because experiments cannot distinguish any microtubule structure, but is also partially based on the experimental observation that axonal bundles are organized in a circular structure for smaller caliber axons^34^. If a vesicle detaches it thus can re-attach to any nearest-neighbor on either side with symmetric equal weighting.

We follow a dynamic Monte Carlo simulation method to determine the vesicle behavior at each simulation time step (see Appendix A.3 for the algorithm). We define our minimum time-resolution window for simulations at 20 ms. The 20 ms limit is below the lower limit time-resolution used in fluorescence microscopy experiments^7,13^, but larger than single-motor stepping dynamics^38^, which allows us to include ATP/ADP dynamics as a single parameter. At the beginning of each simulation, all probability parameters (Pd, Pj) are defined and fixed for the remainder of the simulation. At the beginning of each time step, random numbers are generated from an unweighted distribution. The vesicle is then determined to detach/re-attach, hop, or walk, by comparing the random numbers to their corresponding probability values.

#### Modelling the kymograph representation

The SVP mobility experiments presented above (Fig. 1) utilize a kymograph representation of experimental fluorescence microscopy images. The kymograph representation transposes two-dimensional intensity data onto a single one-dimensional line. Kymographs provide the ability to observe low-intensity events due to experimental imaging limitations in live-cells. However, as a consequence, kymographs cannot distinguish individual tracks within a bundle. We compare our model to experimental results by making an equivalent one-dimensional reduction of our two-dimensional simulation.

We reduce our two-dimensional model by representing each aligned lattice site on a track as a single one-dimensional lattice site (Fig. 4B). All two-dimensional lattice sites along individual tracks are aligned along the defined y-direction (Fig. 4A). If a vesicle is on any track in the bundle at a specific lattice site, then it is represented as along a single equivalent one-dimensional lattice site. This simplification preserves the original two-dimensional simulation results and spatial-resolution, while following the experimental kymograph method of representation. We also introduce a new parameter to account for bundle information lost due to experimental resolution limits, which we define as inhibition probability (P_h_) to model the probability a cargo will pause at a MT-end (Vertical line in Fig. 4B). The inhibition probability is mathematically defined as the number of microtubule ends divided by the number of microtubule tracks within the bundle at a lattice site (x). An example simulation shows an equivalent kymograph position as a function of time (Fig. 4C). The location of the vesicle along the x-axis of the bundle track (horizontal-axis) as a function of time also shows a pause at a MT-end (straight vertical line in Fig. 4C). The specific track the motor traverses is lost in the kymograph representation but pausing behavior at MT-ends is preserved.

#### Determining length of time an SVP pauses using pause-time distributions

To compare our model to experimental observations we focus exclusively on x-axis positions that include microtubule ends (the yellow dashed box in Fig. 4A). In this approach, the MT-end x-axis positions are identified and the time any simulated cargo spends at that position is extracted. All times of all vesicles are binned in a histogram and normalized to obtain an ensemble distribution.

To accurately quantify and compare our model to experiment, we present a parameter that we call pause time (PT). The PT parameter measures the probability an ***aggregate distribution*** of vesicles will pause near a MT-end site in a bundle, similar to a previously developed observable used to describe single kinesin-1 motor motility on single microtubules with obstructions^11^. The PT parameter does not measure the time on a specific microtubule, but quantifies how long a vesicle pauses at the same x-axis position as a microtubule end within the bundle (Fig. 4B), consistent with experimental limitations^7^.

The advantage of the PT–distribution metric is that it can distinguish if a single rate constant (Pd) is sufficient to model how vesicles navigate MT-ends (see Appendix A.2 for derivations). First, vesicles that do not pause at a MT-end are only observed for one time-step/frame. Second, vesicles that pause have a probability to pause based on the pause-duration and inhibition probability. Both are represented mathematically as:

1 $$\begin{aligned} \rho \left( {t = 1} \right) & = 1 - P_{h} \\ \rho \left( {t > 1} \right) & = P_{h} [\left( {1 - P_{d} } \right) + P_{d} P_{h} ]^{t} P_{d} \left( {1 - P_{h} } \right) \\ \end{aligned}$$

where *t* is in the units of the time-step/exposure interval and the probability of any specific vesicle pausing at an MT-end is P_h_ ≈ 1/N_track_, which assumes all tracks are equally accessible in the bundle. The number of time steps paused at the MT-end is denoted by t. This equation is also derived from a simple probabilistic model described in Appendix A.3.

An example distribution of simulated vesicles paused at a MT-end shows how to quantitatively determine a single rate constant model (Black squares in Fig. 4D). The probability that an SVP will pause (y-axis) decreases with increasing time (x-axis) for a simulated distribution. The single rate-constant probability model (Eq. 1) reproduces the distribution and provides specific testable parameters. We will use *both* Eq. 1 and computational models to distinguish how our model can explain the experimental results.

Lastly, to highlight the qualitative nature of an aggregate SVP pause-time at MT-ends we modeled pause-duration probability of P_d_ = 0.03 per 20 ms time-step, equivalent to a pause-duration rate-constant ~ 1.5 s^−1^ (Black squares Fig. 4E). The initial pause-time (t = 0) represents the fraction of SVP that traverse the MT-end location. The remaining fraction of SVP pauses decreases with increasing pause-time (t′).

### Pause-time distributions for retrograde motion:

**Figure 5:**
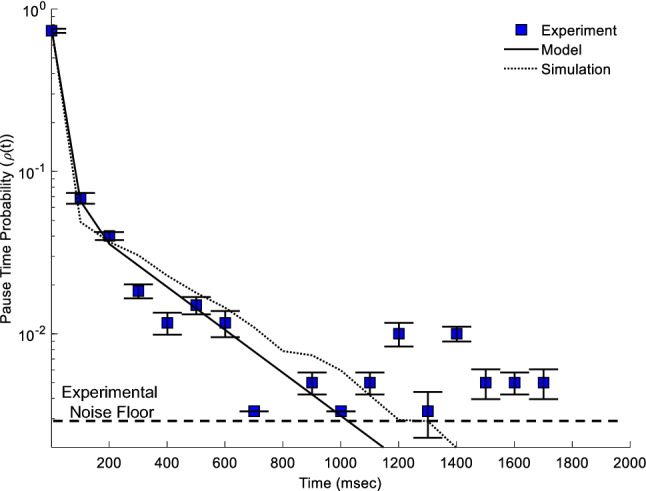
Retrograde-driven transport pause distribution as a function of time. The SVP traverse fraction (t = 0 ms) is included along with the model fit at that data point for context. Experimental data shows a decrease in pause-time probability with increasing time (Blue squares, N = 599. Both the analytical model (Eq. 1, solid line, Pd = 0.35) and the coarse-grained computational simulation (dashed line, Pd = 0.35) reproduce the observed experimental results. The lower-limit of pause-time fraction used to fit to the model was the gaussian random noise (1/Number of SVPs, “Experimental Noise Floor”, dashed line).

Retrograde driven transport pausing at MT-ends exhibits a decreasing probability with exposure time. We used the same locations as measured for SVP traverse fractions (Fig. 2), with the traverse fraction plotted as the intercept of the pause-time distribution (y-intercept Fig. 5). We aggregated the remaining pauses of the 599 events as a single distribution (Blue Squares, see Appendix 1 for method). The total probability distribution that retrograde driven vesicles will pause at any given MT-end was summed to be (0.24 ± 0.02, or 1—traverse-fraction). The probability of pausing for a specific amount of time decreased from 0.06 ± 0.005 for 110 ms to 0.0017 ± 0.0017 for any time longer than 1 s (where the longer-time values is limited by the total number of events 1/599 = 0.0017). This decreasing pause-time distribution suggests that a single rate-constant determines the time any given vesicle will pause at a given MT-end. We note that multiple rate-processes may exist, but a two-rate model does not improve the qualitative or quantitative results compared to a single rate model (See Appendix A7).

We modeled the retrograde probability distribution based on our SVP single pause-duration model (see Sect. 2.4 and Appendix A2). We set the vesicle traverse fraction in our model to the closest number integer MTs (N = 4) that fit the observed fraction in Fig. 2. We then determined the best parameter fit of our model to the data using un-weighted chi-squared analysis (See Appendix A6). Our model shows that the vesicle pause distribution follows a pause-duration probability of 0.375 per 110 ms (solid black line in Fig. 5).

We computationally simulated vesicle mobility on a lattice with a single pause-duration probability parameter per time-step (see Sect. 3.4, and Appendix 4 for model details). We reproduced experimental results by first modeling smaller time/space resolutions (24 nm spatial and 20 ms time resolution), followed by coarse-graining simulated results similar to the way camera exposure frames average. This coarse-graining approach results in the same general behavior, but increased variance (see Appendix A3, A5). We simulated vesicles with a Pd = 0.07 per 20 ms time-steps, which is equivalent to the experimental Pd = 0.35 for 100 ms exposure-time (where 0.35 = 0.07*100msec/20msec). This simulated pause-duration reproduced the experimentally observed pause-time distribution (dashed line). The difference between analytical and computational simulations are due to the slight differences caused by coarse-graining simulations (Appendix A5).

Both the analytical and computational models show that a single rate constant model accurately reproduces the majority of observed vesicle pause-times at MT-ends. We attempted to fit the longer pause-time pauses (> 1 s) with a model that assumes two possible distributions of pausing vesicles (See Appendix A7 for model); however, this two-rate model did not significantly improve the quality of fit, or the quantitative value of fit (Appendix A7). This is most likely because not enough vesicles were measured to accurately determine longer pause-times. Thus, a single constant Pd = 0.35 captures that majority of the observed pause-time distribution. This result thus suggests that a single pause-rate time-scale determines how long vesicles will pause at MT-ends during transport.

### Pause-time distributions for anterograde motion

It is well known that retrograde and anterograde motion utilize fundamentally different motors to drive transport^39^. Further, different end-binding proteins localize at the minus or plus end of MTs^40^. Consequently, it may be possible that retrograde and anterograde motion follow three following pathways:

(i) the difference in SVP dissociation/association rates alone result in different mechanics at MT-ends. The difference in mechanics would result in fundamentally different observed pause-time distribution results.
(ii) all the same motor/protein complexes co-bind on SVPs and the MT to help navigate MT-ends, *but not result in a reversal,* which would result in a single pause-time distribution *regardless* of direction of travel.
(iii) different proteins at plus/minus MT-ends may mediate SVP navigation of MT-ends and dictate vesicle pause-duration, regardless of motor driven transport. These different proteins would then result in different pause-durations for anterograde and retrograde motion. Further, this possibility would be distinguished from (i) if vesicle pause-durations do not match individual motor dissociation/association rate-constants.

We sought to test these three hypotheses by quantifying the pause-time distribution for anterograde motion separate from retrograde motion.

Anterograde driven transport pausing at MT-ends exhibits a continuously decreasing probability with time. We calculated the distribution of Anterograde pausing at MT-ends by aggregating pauses of 737 events from 34 different MT-ends in 8 different experiments (see Appendix A1). The probability of pausing for a specific amount of time decreased from 0.1 ± 0.005 for 110 ms to 0.0012 ± 0.0012 for any time longer than 1 s (where the longer-time values is limited by the total number of events 1/737 = 0.0012).

The pause-time distribution follows the same general reduction as a function of time observed for retrograde driven transport. This suggests that our overall single rate-constant model can describe both directions of motion. Further, anterograde and retrograde driven pause-time distributions are significantly different from each other. However, we must still model anterograde pause-times in order to distinguish between hypotheses (i) and (iii).

**Figure 6:**
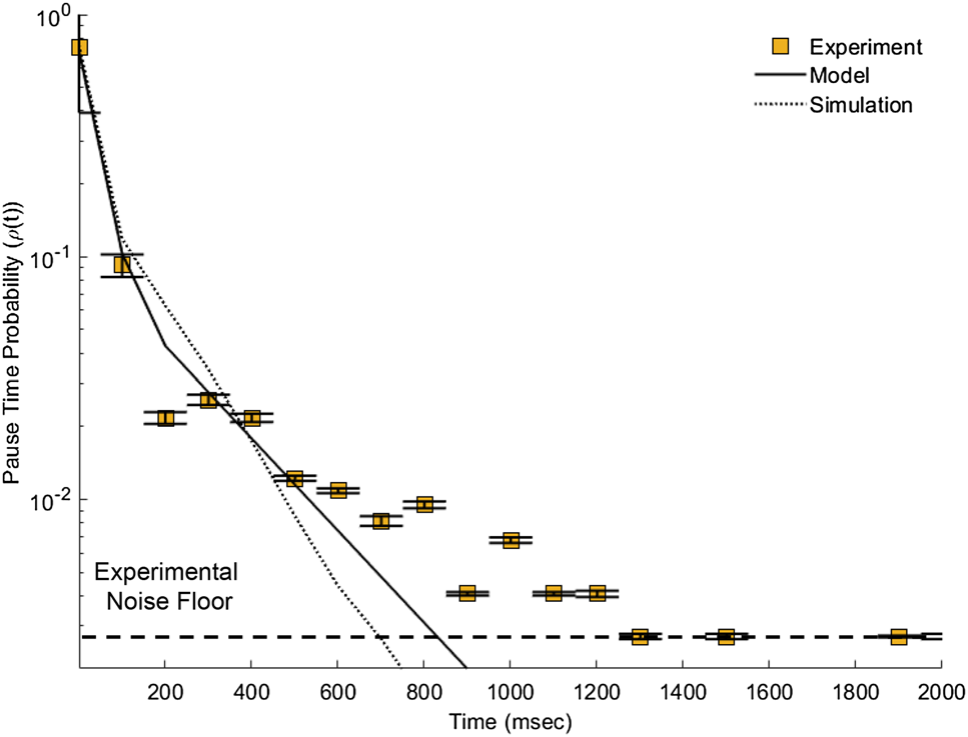
Anterograde-driven transport pause distribution as a function of time. The SVP traverse fraction (t = 0 ms) is included along with the model fit at that data point for context. Experimental data shows a decrease in pause-time probability with increasing time (Yellow squares, N = 737). Both the analytical model (Eq. 1, solid line, Pd = 0.5) and the coarse-grained computational simulation (dashed line, Pd = 0.65) reproduce the observed experimental results. The lower-limit of pause-time fraction used to fit to the model was the gaussian random noise (1/Number of SVPs, “Experimental Noise Floor”, dashed line).

We modeled the anterograde probability distribution based on the same attachment/detachment mechanics (See Appendix A2). We set the vesicle traverse fraction in our model to the observed fraction in Fig. 2. We set the vesicle traverse fraction in our model to the closest number integer MTs (N = 4) that fit the observed fraction in Fig. 2. We then determined the best parameter fit of our model to the data using un-weighted chi-squared analysis (See Appendix A6). Our model shows that the vesicle pause-time distribution follows a pause-duration of 0.5 per 110 ms (solid black line in Fig. 6). Biasing our model toward earlier pause-times or later pause times did not significantly improve the fit to data (Appendix A6). Thus, a single constant P_d_ = 0.5 ± 0.1 captures that majority of the observed pause-time distribution.

We computationally simulated vesicle mobility for anterograde motion exactly the same as with retrograde motion (discussed above) but with a different pause-duration probability (see Sect. 3.4, and Appendix A2—A4 for model details). Our simulated vesicles with a P_d_ = 0.13 (equivalent to an experimental P_d_ = 0.65 in 110 ms coarse-grained time) reproduced experimentally observed pause-time distribution (dashed line). The difference between analytical and computational simulations are due to the slight differences caused by coarse-graining simulations.

The difference between anterograde and retrograde pause-time distributions, combined with the difference in time-scales between pause-duration and single motor association/dissociation times, suggests that SVP pause-times are determined by different proteins interacting with the vesicle at the time it reaches a MT-end (hypothesis (iii)). Further, retrograde driven motion has a lower pause-duration probability than anterograde driven motion (Pd = 0.35 for retrograde, and Pd = 0.5 for anterograde). This difference is consistent with previous observations of pausing at MT-ends^14,20–22^.

## Conclusions

We have quantified the pausing behavior of synaptic vesicle precursors at microtubule-end locations (MT-end) in vivo. We showed that vesicles have the same probability of being stopped at MT-end locations for both anterograde and retrograde motion. We also showed that vesicle reversals are a small fraction of motion after vesicles leave MT-end locations, suggesting that tug-of-war mechanics are a minority of behavior. We propose a single-rate model by which vesicles navigate MT-ends, based on the observation that vesicles pause for different times depending on direction of motion before reaching a MT-end. We show that this model reproduces both anterograde and retrograde pause-time distributions. We also computationally simulate vesicles at MT-ends with a single-rate pause-duration parameter and show that the simulations reproduce observed experimental pause-times.

## Discussion

The results in this study show that the direction of travel at the time the vesicle reaches a MT-end dominates how long the vesicle pauses before continuing its motion. Combined with the low reversal fraction at MT-ends, this direction dependent pausing suggests that vesicles rely on mechanics of the driving motor in combination with other possible co-binding proteins to navigate MT-ends. Our pause-time analysis and single rate-constant result is significant because different mechanisms can occur simultaneously affecting the qualitative nature of the pause-time distribution. For example, a UNC-104 motor mutation, which drive anterograde motion, may result in sub-populations of longer vesicle pausing at MT-ends, which in our model would be described by more than one rate-constant, but not result in a change in the average pause-time; any interpretation based on average pause-time alone would miss the difference in mechanics observed by measuring pause-time distributions. Thus, measuring the pause-time distribution and comparing to a rate-constant model provides increased understanding of the underlying mechanics. Future experiments that modify either molecular motor mechanics or MT-end binding proteins should compare SVP pause-time distributions to a single-rate constant model in order to further determine the validity of this paradigm.

Our current results show that a single rate-constant model describes vesicle pause behavior at MT-ends but does not distinguish if more than one protein is involved in the process. It is well established that multiple protein process pathways can work in parallel resulting in a single observed rate-constant^41^. While the difference in average rate can distinguish that different mechanics exist for retrograde and anterograde motion, but average rate-constant result *alone* cannot distinguish if multiple proteins coordinate at MT-ends. Our model can thus provide a quantitative basis to distinguish effects on both the SVP pause-time and mechanics of pausing at MT-ends. For example, if a minus-end binding protein reduces pause times by forcing SVPs to dissociate faster then a mutation in this protein could result in *both* a larger average pause time and a deviation from a single rate-constant behavior.

Many cell signaling processes rely on transport of signals from the periphery of the axon to the cell nucleus, or vice versa with a signal sent from the cell nucleus to the periphery. Vesicles must navigate any MT-end they encounter during that process. While each pause at a MT-end does not contribute a significant amount to the overall transport time, accumulating multiple vesicle pauses along the axon can contribute significantly to transport times. Further, if a signaling process requires tightly controlled transport times then any deviation can result in altered cellular behavior. *The results in this study show that the mechanics of the driving motor are important in distinguishing the amount of time navigating MT-ends contributes to overall transport time*.

## Experimental methods

Raw data collection was performed as previously described^7^. Briefly, young-adult hermaphrodite *C.elegans* expressing the synaptic vesicle precursor marker RAB-3 in the DA9 motor neuron (wyIs251[Pmig-13::GFP::RAB-3]) were paralyzed in 0.3 mM Levamisole in M9. Once paralyzed, worms were carefully transferred to M9 solution on a 10% agarose pad for imaging. This results in an effective Levamisole concentration which is significantly lower than the concentration that was suggested to affect axonal transport^42^. Worms were maintained on the pad for no more than 20 min, although we confirmed that viability was maintained even after 4 h.

Fluorescence imaging was performed using a Nikon 60 × CFI plan Apo VC, NA 1.4 objective on a Nikon Ti-E microscope equipped with Yokogawa CSU-X1 scan-head and a Hamamatsu C9100-50 EM-CCD camera at a frame rate of 110 ms/frame, and 240 nm per pixel.

Post-analysis fluorescence movies were corrected and analyzed in imageJ. Animal movement was corrected using FIJI plugin StackReg. Kymographs were generated with KymoBuider. Intensity was averaged ± 5 pixels transverse to the kymograph line.

## Supplementary Information

Supplementary Information 1.

Supplementary Video 1.
