# Supplementary material for: Distinguishing synaptic vesicle precursor navigation of microtubule ends with a single rate constant model
Source: Sci Rep. 2021 Feb 9;11:3444. doi: 10.1038/s41598-021-82836-7 (PMC7873188; doi:10.1038/s41598-021-82836-7)
Supplement: Supplementary file 1 — Supplementary Information 1. [file 41598_2021_82836_MOESM1_ESM.docx]

**Supplementary Material**

**Title:** Distinguishing Synaptic Vesicle Precursor Navigation of Microtubule Ends With A Single Rate Constant Model

**Authors:** M.W. Gramlich*^1^, S. Balseiro Gómez^2^, S. M. Ali Tabei ^3^, M. Parkes ^1^, S. Yogev ^2^

1. Department of Physics – Auburn University

2. Departments of Neuroscience and Cell Biology – Yale School of Medicine

3. Department of Physics – University of Northern Iowa

* Corresponding Author: [mwg0016@auburn.edu](mailto:mwg0016@auburn.edu)

**Appendix A1: Data Aggregation and error methods**

We quantified aggregate pause-time distribution for MT-ends by combining all motor pauses into a single binned distribution using the following method (presented as an example in Table A1 below):

[i] We binned cargo based on the number of frames they were observed at the x-position along the kymograph such that: cargo that traversed a MT-end were observed for 1-frame, cargo that paused for 1-exposure-time were observed for 2-frames, etc …

[ii] We distinguished anterograde and retrograde cargo motion and counted them separate bins.

[iii] We performed [i] and [ii] for 15 different MT-end locations across 4 different experiments.

[iv] We then summed the individual bins into a single final bin as follows: all cargo that traverse MT-end locations (defined as bin = 1-frame discussed in [i]) were summed together for a total of 288 tracks for anterograde and 271 tracks for retrograde; all cargo that paused for a single exposure-time (defined as bin = 2-frames discussed in [i]) were summed together for a total of 39 tracks anterograde and 23 tracks for retrograde; etc..

[v] The number of vesicles in each bin are then normalized by the total number of tracks 359 tracks anterograde and 388 tracks for retrograde;

[vi] The resulting distributions are plotted in Fig. 5 for retrograde and Fig. 6 for anterograde

[vii] Errors were calculated as the standard-deviation in number of vesicles across all MT-ends, for a given bin (n = 1, 2, 3, …) divided by the total number of vesicles observed.

Table A1: Anterograde pause distribution Aggregation Method Example

Figure A1: Anterograde pause distribution Aggregation Histogram

**Appendix A2: Derivation of Probabilistic Model of Motor Pause Distributions at Microtubule ends:**

Figure A2: Analytical Probability Model.

(A) The probability a motor pauses at a site with an obstruction (P_h_) is equal to the number of ends divided by the number of tracks.

(B) The probability a motor waits a single time step at an obstruction is the combined probability of not detaching and the probability that a motor detaches and re-attaches to the same track.

(C) The probability a motor leaves in a time step is the probability that a motor detaches, re-attaches, and occupies a new track.

We now derive the analytical model of single motor pausing at microtubule ends *in the one-dimensional simplification*. This derivation works from the experimental perspective that we do not know the individual microtubule track a motor is on. Further, the derivation is based on a combinatorial approach to a one-dimensional experimental observation. At each time-step a motor may execute a combination of different processes each one dependent upon the other in the following ways:

1. The first time a motor reaches a microtubule end position there is probability it may ***not*** be on the same microtubule track as the microtubule end (1 – P_h_), and thus ***not pause***.
2. If the motor is on a microtubule track with a microtubule end then it will pause for its first time-step at that position (P_h_), as shown in Fig. A.2A.
3. If a motor has paused from condition (ii) then there are two ways it may continue to pause (Fig. A.2B):
   - - - 1. The motor doesn’t detach (1 – P_d_)
         2. The motor does detach, re-attach, and is still on the same track as the microtubule end (P_d_ P_h_)
4. The motor will continue testing condition (iii) above for (t) time-steps, with each step multiplied by the previous number of attempts
5. At (t) time-steps a motor detaches and re-attaches to a different microtubule track than the microtubule end (P_d_ (1 – P_h_)), as shown in Fig. A.2C.

The first process above (i) is simply the number of motors that do not pause at a known microtubule end site. The following processes (ii)-(iv) are the number of possible ways motors may test a microtubule end site and combine into eqn. 1. We have included the time required for a motor to re-attach to another microtubule track (iv) because this would be how experiments would determine if a motor has been able to traverse a microtubule end.

**Appendix A3: Vesicle Simulation Algorithm:**

Figure A3: Simulated Vesicle Algorithm Flow Chart

The vesicle decisions at each time step (ti) follow a specific algorithm, shown in Fig. A3. At the beginning of each simulation, a bundle generated from the bundle algorithm is loaded from a file into a two-dimensional array U. The motor simulation then uses the fixed obstruction locations for the remainder of the simulation.

First, three random numbers (R1, R2, R3) are chosen at the beginning of each time step, chosen from an unweighted python number generator random.rand(), which uses a Mersenne Twister algorithm to generate random numbers between [0, 1). Following the dynamic Monte Carlo approach, all random numbers are discarded at the end of each time step and a new set of random numbers is chosen at the next time step.

Second, vesicle detachment, re-attachment, and microtubule-switching tests were run, shown in Fig. A3. A motor detaches if R1<Pd, otherwise the obstruction test is run. If a vesicle detaches, then it re-attaches in the same time-step.

Third, a re-attached vesicle randomly chooses a microtubule track to hop on, determined by the random number R3, with all tracks equally likely to be chosen. The algorithm to determine a specific track is as follows: the probability for all tracks are assigned a decimal range (i.e. Track 1 P1= [0,1/Ntrack), Track 2 P2= [2/Ntrack,1/Ntrack)), a vesicle reattaches to a track if R3 is within the assigned range (i.e., if R3 ϵ P1, then the motor is attached to Track 1).

Once motor-bundle interactions have been determined, a motor-obstructions test is run, shown in Fig.A2. If there is no motor obstruction at the next lattice site to be occupied by the motor, then the motor moves to that site. If there is an obstruction at the next lattice site, then the motor remains on the same lattice site for the next time step.

The simulation ends if the motor reaches the end of the bundle or the time steps exceed a maximum threshold. In the case of perfectly aligned bundles, motors always reach the end of the bundle. In the case of completely unbiased bundles, motors may not reach the end of the bundle depending on the number of obstructions and their respective inhibition probability. Therefore, a maximum threshold of 10,000 time steps was set.

Notably, alternative algorithms, such as the Gillespie Algorithm, have been used to model single-motor motility. However, these algorithms would result in the equivalent measurable quantities to those described in this manuscript. Our interest is to provide an algorithm that mimics the randomness at the local time step level, which we can easily achieve with dynamic Monte Carlo simulation.

**Appendix A4: Coarse-Graining Method**

Vesicles were coarse-grained (CG) after 2D bundle motility simulation and a 1D simplification. Simulations were CG by defining a new lattice site, which is an integer number of un-coarse-grained lattice sites (ΔX = n*Δx). The time in the CG lattice site is calculated as the difference in time a motor spends within the n-lattice sites, i.e., the CG simulation time for each ΔX lattice site. The CG inhibition probability (Ph) is the simple sum of all Ph values within the n-lattice sites, which is based on the assumption that each time a motor encounters a Ph, it has no memory of any previous microtubule end.

It is helpful to distinguish different conditions that lead to different CG simulation times at each CG lattice site to better understand how they may be observed experimentally.

For the completely biased bundle, there are two conditions:

(i) A motor hops through all un-CG lattice sites without obstructions and the CG time is ΔX/Δx;

(ii) A motor pauses at one or more un-CG lattice microtubule end sites within a single CG-lattice site, resulting in a CG-time of ΔX/Δx + Pause-Times at microtubule ends. Note that the Ph is the sum of all microtubule ends within a CG-lattice site and thus corresponds to the increased CG time.

We note that simulation time steps and CG time can be converted to the Lab-time. In the un-CG simulation, a single time step is 30 msec. CG time steps are integer multiples of un-CG time-steps (n*30 msec, n = 1, 2, 3, etc.). Thus, the Lab-times used in this simulation are integer multiples of 30 msec.

**Appendix A5: Pause Time Measurements Depend on Camera Exposure Times**

Figure A4: Pause-time distributions must be determined in Lab-time:

(A) Pause-time distributions are calculated for 1000 simulations of vesicles with Pd = 0.03 per time-step equivalent to 30 msec/frame camera exposure (Blue triangles). The same simulated vesicles were coarse-grained by 2-frames (Red squares) and 5-frames (Green squares). Plotting the pause-time distributions on time-step scales appears to change the distributions.

(B) The same pause-time distributions in (A) converted to lab-time. All distributions show the same qualitatively equivalent distributions, but with different density of points.

The resolution of the experimental camera exposure time affects the ability to measure PT. This phenomenon has been of considerable interest in many single-molecule experiments focusing on improving instrumentation ^1,2^ and data analysis techniques ^3^. More recently, there has been interest in de-convolving real rate-processes from the camera response at or near the single-frame resolution limit ^4^. Considering the interest in such resolution limitations, we show that Pause-time distributions must be measured in lab-time frames rather than experimental camera exposure time.

We use a single bundle simulation with vesicles that have the same detachment (P_d_ = 0.03). After the simulations, we calculated the one-dimensional bundle (See Section 3.4). We then coarse-grained the one-dimensional results by combining a number of x-lattice sites (1, 2, 5 bins, see appendix A3 for algorithm). We then correlate the increased spatial limit with increase time resolution (1, 2, 5 time steps) to represent different camera exposure times (30, 60, and 150 msec). This method mixes time/space resolution limits, which is consistent with experimental experiments.

**Appendix A6: Chi-squared analysis of model versus data**

| Pd | Anterograde | Retrograde |
| --- | --- | --- |
| 0.1 | 8.21E-03 | 3.10E-03 |
| 0.2 | 5.14E-03 | 1.48E-03 |
| 0.25 | 4.05E-03 | 1.01E-03 |
| 0.275 | 3.60E-03 | 8.48E-04 |
| 0.3 | 3.20E-03 | 7.34E-04 |
| 0.325 | 2.84E-03 | 6.67E-04 |
| 0.375 | 2.28E-03 | 6.71E-04 |
| 0.4 | 2.06E-03 | 7.43E-04 |
| 0.5 | 1.65E-03 | 1.50E-03 |
| 0.525 | 1.66E-03 | 1.81E-03 |
| 0.55 | 1.71E-03 | 2.17E-03 |
| 0.575 | 1.81E-03 | 2.58E-03 |
| 0.6 | 1.95E-03 | 3.04E-03 |
| 0.625 | 2.14E-03 | 3.54E-03 |
| 0.65 | 2.38E-03 | 4.10E-03 |
| 0.675 | 2.66E-03 | 4.70E-03 |
| 0.7 | 2.99E-03 | 5.36E-03 |

Table A2: Chi-square values for model/data comparisons

**Appendix A7: Two-rate Model Fit to Data**

We fit the observed anterograde pause-time distribution to a two-rate model to improve the quality of fit to longer pause-times. We observed that our single-rate model reproduced early time pauses but did not match the longer-time pauses (Fig. 5, 6). We hypothesized that the observed pause-time distribution may be a collection of more than one population of vesicle pause-durations. If this hypothesis is true, then the observed pause-time distribution may be best fit with a model that includes a mixture of two different pause-duration parameters.

We tested this hypothesis by making a two-rate model to fit to the anterograde pause-time distribution. We created our two-rate model as follows:

1. We made two separate models (ρ_1_, ρ_2_), each described by eqn. A1 in section 3.4.3, with a single fixed pause-duration (P_d1_, P_d2_). For example, we made two models with P_d1_ = 0.1 and P_d2_ = 0.7.
2. We then added a variable parameter (φ) that determined the fraction that each model would contribute in the two-rate fit, but constrained to a combined fraction summed to 1.
3. We then summed the fractional contribution from each model. This fractional sum was then compared to the observed pause-time distribution.
4. Finally, we used a chi-square analysis to determine the best fraction value that would fit given the two pause-duration values.

This two-model fit is best described mathematically as:

$\rho\left( t,\phi,P_{d1},P_{d2} \right)={\phi*\rho}_{1}\left( t,P_{d1} \right)+(1-\phi)*\rho_{2}\left( t,P_{d2} \right)$ {A.2}

Figure A5: Anterograde pause distribution fit with a mixed model.

(A) Chi-Squared analysis of different model fits to anterograde data. Mixed models were created as a fraction of two different pause-duration parameters (See text in Appendix A7), calculated based on the analytical model in Appendix A3. The chi-squared values were calculated as a function of the fraction parameter in the model (φ, x-axis).

(B) The lowest chi-square fits for each mixed model are plotted as compared to experimental data (Same line/symbols used as in A). No mixed model significantly improves the quality of fit as compared to a single pause-duration parameter model.

We fit the two-rate model described above to observed anterograde pause-time distribution (Fig. A5). We created a combination of different pause-duration values with a low-value (P_d1_) and a high-value (P_d2_). We varied the fraction of each low/high value and compared the combined two-rate model to observed data using the same unweighted chi-squared analysis for the single-rate model. Finally, we plotted the different chi-squared fits as a function of the fraction-parameter (Fig. A5 A).

No two-rate model significantly improved the chi-squared fit as compared to the single-rate model fit (Fig. A5 A). The lowest chi-square value for each two-rate fit matched the single-rate fit (solid horizonal line). The best two-model fit (P_d1_ = 0.2/ P_d2_ = 0.8) gave ~13% improved fit over the single-rate model. Further, the two-rate model (P_d1_ = 0.3/ P_d2_ = 0.4) gave the worst chi-squared fit, with the best chi-squared occurring when the model is entirely the single-rate of 0.4 (φ = 0); this is likely due to both pause-duration parameters below the 0.5 value for the single-rate model. *These results show that a two-rate model does not significantly improves the quantitative fit to observed anterograde pause-time distribution.*

The two-rate model slightly improved the qualitative fit as compared to the single-rate model (Fig. A5 B). We compared the best-fit pause-time distributions for different two-rate models, obtained using chi-square analysis above (Fig. A5 A), to the observed anterograde pause-time distribution (Squares Fig. A5 B). We also compared the two-rate models with the best-fit single-rate model (P_d_ = 0.5, Solid line Fig. A5 B). *All two-rate models slightly improved the quality of fit to longer-time pauses but at the expense of worse fits to short-time pauses*.

**Appendix A8: SVP pause duration at random MT locations are significantly different**

Figure A6: SVP pause distributions at MT-ends and random locations.

(A) A direct comparison of Anterograde SVP pause-distributions at random MT locations (Red circles) and MT-end locations (Squares).

(B) A direct comparison of Retrograde SVP pause-distributions at random MT locations (Red circles) and MT-end locations (Squares).

We quantified the pause-time distribution of SVP at random locations along the MT-bundle as a control for pausing at MT-end locations. We randomly chose 13 different locations along the same MT-bundles that were used for MT-end analysis. We required that the random site locations did not have a SVP paused at the beginning of the movie. We also required at least 10 SVPs co-localize at the random sites in order to keep the same statistical basis as MT-end locations. We otherwise did not put any restrictions on the number of SVPs that traversed or paused at these random sites. We counted the number of SVPs that traversed or paused at these locations following the same approach outlined in appendix A1, and then aggregated the results (described in appendix A1). We found that SVPs traversed/paused these locations 393 times during anterograde motion and 353 times during retrograde motion.

We found that both anterograde and retrograde pause-time distributions were significantly different than compared to SVP pause-time distributions at MT-end locations (Fig. A6). The majority of SVPs traversed these locations, with more traversals than observed at MT-end locations (Fig. 2 main text). Further, there were fewer reversals at these random locations than at MT-end locations (Fig. 3 main text). Both anterograde and retrograde distributions exhibited an overall lower probability as compared to SVP pauses at MT-end locations.

**Appendix A9: SVP Anterograde and Retrograde pause duration at MT-ends are statistically significantly different**

Figure A7: SVP cumulative pause distributions at MT-ends versus random sites. (A) Cumulative plots comparing Anterograde pause distributions at MT-ends (circles) as compared to random sites (solid line). (B) Cumulative plots comparing Retrograde pause distributions at MT-ends (circles) as compared to random sites (solid line). (C) Cumulative distributions comparing anterograde and retrograde pause distributions at MT-end sites. (D) Model fit-values (P_d_) for retrograde and anterograde pause-distribution fits. The cumulative distributions are statistically significantly different as determined by two-tailed KS-test, ** p < 0.01, *** p < 0.001

We determined the statistical significance of retrograde and anterograde distributions for MT-end sites as compared to random sites (Fig. A7 A,B) as well as comparison between Anterograde/Retrograde at MT-end sites (Fig. 7 C,D) using cumulative distributions to distinguish small differences in the pause distributions. The retrograde pause distribution shows a clear and statistically significant difference (p = 0.0044) from anterograde pause distribution.

**Appendix A10: Supplementary Movie of Raw data**

A movie of SVP traversing the axon is shown in SM1. Multiple SVPs can be seen traveling along the axon.

**Supplementary References**

1. Beausang, J. F., Shroder, D. Y., Nelson, P. C. & Goldman, Y. E. Tilting and Wobble of Myosin V by High-Speed Single-Molecule Polarized Fluorescence Microscopy. *Biophysical Journal* **104**, 1263–1273 (2013).

2. Verbrugge, S., Kapitein, L. C. & Peterman, E. J. G. Kinesin Moving through the Spotlight: Single-Motor Fluorescence Microscopy with Submillisecond Time Resolution. *Biophysical Journal* **92**, 2536–2545 (2007).

3. Woody, M. S., Lewis, J. H., Greenberg, M. J., Goldman, Y. E. & Ostap, E. M. MEMLET: An Easy-to-Use Tool for Data Fitting and Model Comparison Using Maximum-Likelihood Estimation. *Biophysical Journal* **111**, 273–282 (2016).

4. Lewis, J. H., Jamiolkowski, R. M., Woody, M. S., Ostap, E. M. & Goldman, Y. E. Deconvolution of Camera Instrument Response Functions. *Biophysical Journal* **112**, 1214–1220 (2017).
